# Prediction of mortality in very low birth weight neonates in Spain

**DOI:** 10.1371/journal.pone.0235794

**Published:** 2020-07-09

**Authors:** Martín Iriondo, Marta Thio, Ruth del Río, Benjamin J. Baucells, Mattia Bosio, Josep Figueras-Aloy

**Affiliations:** 1 Neonatology Department, Hospital Sant Joan de Déu, BCNatal, Hospital Sant Joan de Déu-Hospital, Barcelona University, Barcelona, Spain; 2 Newborn Research Centre, The Royal Women's Hospital, Melbourne & University of Melbourne, Melbourne, Australia; 3 Murdoch Childrens Research Institute, Melbourne, Australia; 4 University of Melbourne, Melbourne, Australia; 5 Barcelona Supercomputing Center (BSC), Barcelona, Spain; 6 Neonatology Department, Hospital Clínic, BCNatal, Hospital Clínic- Hospital Sant Joan de Déu, Barcelona University, Barcelona, Spain; University of Alberta, CANADA

## Abstract

**Objective:**

Predictive models for preterm infant mortality have been developed internationally, albeit not valid for all populations. This study aimed to develop and validate different mortality predictive models, using Spanish data, to be applicable to centers with similar morbidity and mortality.

**Methods:**

Infants born alive, admitted to NICU (BW<1500 g or GA<30 w), and registered in the SEN1500 database, were included. There were two time periods; development of the predictive models (2009–2012) and validation (2013–2015). Three models were produced; prenatal (1), first 24 hours of life (2), and whilst admitted (3). For the statistical analysis, hospital mortality was the dependent variable. Significant variables were used in multivariable regression models. Specificity, sensitivity, accuracy, and area under the curve (AUC), for all models, were calculated.

**Results:**

Out of 14953 included newborns, 2015 died; 373 (18.5%) in their first 24 hours, 1315 (65.3%) during the first month, and 327 (16.2%) thereafter, before discharge. In the development stage, mortality prediction AUC was 0.834 (95% CI: 0.822–0.846) (p<0.001) in model 1 and 0.872 (95% CI: 0.860–0.884) (p<0.001) in model 2. Model 3’s AUC was 0.989 (95% CI: 0.983–0.996) (p<0.001) and 0.942 (95% CI: 0.929–0.956) (p<0.001) during the 0–30 and >30 days of life, respectively. During validation, models 1 and 2 showed moderate concordance, whilst that of model 3 was good.

**Conclusion:**

Using dynamic models to predict individual mortality can improve outcome estimations. Development of models in the prenatal period, first 24 hours, and during hospital admission, cover key stages of mortality prediction in preterm infants.

## Introduction

Preterm birth is the main cause of perinatal mortality and accounts for more than 50% of child disability [1]. Extremely preterm infants (gestational age (GA) under 28 weeks’ gestation), and especially those infants born at the limits of viability (GA between 22 and 25 weeks’ gestation), are the most vulnerable infants in neonatal intensive care units (NICUs), with high morbidity and mortality rates. Decisions regarding care and treatment of these infants are especially challenging, both for health care professionals and families.

Predictive models estimate the probability, or risk, of a certain condition occurring in an individual after the combination of different prognostic factors [2]. They are of utmost importance for complex medical situations with high mortality rates. As reviewed by Medlock et al [3], at least fifty predictive models for preterm infant mortality have been developed. Some, such as the CRIB score [4] or the NICHD model [5], are widely used in neonatal units throughout the world. However, about ten models were developed in the pre-surfactant era [3,4,6] and very few have been externally validated in more than one study [4,5,7].

In Spain, around 30000 preterm infants are born each year [8–9]. The Spanish SEN1500 database was created in 2002 and collects data reported by 65 Spanish NICUs. It includes data from over 2500 very low birth weight (VLBW) infants per year, born with a gestational age (GA) under 30 weeks or birth weight (BW) under 1500g [10–11]. Data collected in this database serves as a benchmark for NICUs, allowing local and collaborative quality improvement projects [12–17].

The aim of this study was to develop and validate different mortality predictive models to be used in preterm newborns in Spain.

## Materials and methods

### Population and study centres

Data was obtained from the SEN1500 database: Infants born alive with BW <1500g or GA <30 weeks, and admitted to 65 Spanish NICUs between 2009 and 2015, were included. There were two main study periods: Development of predictive models (data from years 2009–2012) and validation of models (2013–2015). Exclusion criteria were: Fetal or delivery room deaths, major congenital defects, severe chromosomal abnormalities, and patients transferred to another hospital of whom morbidity and mortality data were missing.

The identity of patients and data collection followed strict confidentiality standards. This study was approved by the Spanish Neonatology Society and the Ethics Committee of Sant Joan de Déu Hospital, Barcelona.

### Study variables

The SEN1500 records perinatal, neonatal and follow-up variables. Perinatal and neonatal variables used in this study, as well as main diagnoses, and severity-adjusted diagnoses adapted in accordance with severity criteria, are shown in Table 1.

**Table 1:** Variables and diagnoses used in predictive models of mortality.

Perinatal and Neonatal variables	Diagnoses *(yes / no)*	Severity-adjusted diagnoses criteria *(yes / no)*
• Maternal steroids *(no / partial / full)* • Maternal chorioamnionitis *(yes / no)* • Maternal hypertension (preeclampsia) *(yes / no)* • Multiple pregnancy *(yes / no)* • Gender *(female / male)* • Gestational age *(weeks and days)* • Birth weight *(g)* • Low weight (<p10—Olsen curves—2010) *(yes / no)* • Place of birth *(inborn / outborn*) • Level of care *(level 2 /level 3)* • Type of delivery *(vaginal / caesarean section)*, • Resuscitation in delivery room *(nil*, *positive pressure support*, *intubation*, *chest compressions*, *adrenaline)* • Apgar test 1 minute *(0–10)* • Apgar test 5 minutes *(0–10)* • Admission temperature income *(°C)* • CRIB-1 score • Postnatal corticosteroids *(yes / no)* • Discharge outcome *(home*, *transfer*, *death)*	• Respiratory distress syndrome • Pneumothorax • Patent ductus arteriosus • Necrotizing enterocolitis • Focal gastrointestinal perforation • Early onset sepsis (<72 h) • Late onset sepsis (>72 h) • Intraventricular hemorrhage • Periventricular leukomalacia • Retinopathy of prematurity • Bronchopulmonary dysplasia	• Advanced resuscitation *(intubation*, *chest compressions*, *adrenaline)* •Severe respiratory distress syndrome *(2 doses of surfactant + positive pressure support or 1 dose of surfactant + positive pressure support + FiO*_*2*_*> 0*.*3)* • Severe patent ductus arteriosus *(surgery)* • Severe necrotizing enterocolitis *(drainage or surgery)* • Severe invasive infection *(positive blood culture + shock / hypotension or meningitis)* • Severe retinopathy of prematurity *(laser therapy)* • Severe intraventricular hemorrhage *(grade 3 or 4 or surgery for hydrocephalus)* • Severe periventricular leukomalacia *(cystic)* • Severe bronchopulmonary dysplasia *(oxygen or pressure support at 36 weeks of postmenstrual age)* • Severe pneumothorax *(chest drainage)* • Severe anemia *(red blood cell transfusion)*

### Predictive models and data analysis

#### Development stage

Three predictive mortality models were developed: Model 1 (prenatal), model 2 (first 24 hours of life), and model 3 (prediction during hospital admission).

Non-parametric statistical tests were used to preselect certain features since none of the quantitative variables followed a normal distribution in the training tests. Variables with a significance of p<0.15 in the Chi-square or Mann-Whitney U test were then introduced as covariates in the regression models. Where possible, evaluation of the model with Nagelkerke’s R2 was performed. For all models, cut-off values with the highest Cohen Kappa index [18] in the training set were selected. Model discrimination was assessed using the area under the curve (AUC) and the odds ratio (OR). The Hosmer-Lemeshow test was used to measure model calibration; if p>0.05, the model was considered well-calibrated [19]. The Brier score, which analyses the deviation between predicted and real probabilities uninfluenced by selected cut-off values, was used to test model accuracy; setting values, from 0 for a perfect model to 0.25 for a non-informative model [20,21]. Predictive models were generated using generalized linear model (GLM) classifiers with R 3.5.1 software (Caret package). The output is a maximum-likelihood estimation for the probability of death of each sample, which is calculated with the following formula: Probability of "death" = 1 / (1+e^-z^), where z is the output value of the model. Conversely, survival probability (in percentage) would be 100 –mortality (in percentage).

**Model 1** is a Prenatal predictive model (dependent variable: Hospital mortality. Independent variables: Maternal and perinatal data, Table 1).

**Model 2** is a 24 hours of life predictive model (dependent variable: Hospital mortality. Independent variables: All maternal and perinatal data, including severe respiratory distress syndrome, Table 1).

**Model 3** is a predictive model during hospital admission (dependent variable: Hospital mortality. Independent variables: All maternal, perinatal and neonatal data from Table 1, plus length of stay in NICU). This model is a composition of two sub-models, which are alternatively applied before or after the 30^th^ day of admission. The choice to split the estimation into a two-step model was based on the empirical different nature of symptoms/features affecting patient survival in the first days, or after a long period in the NICU. Length of stay was incorporated as an independent variable to capture the impact of time on patient outcome.

Since we only had cumulative data from the full NICU stay for patients, in order to develop a model with the capability of expressing time-course evolution, we had to run a data simulation during the development stage. Each patient record was multiplied by the number of days they stayed in the NICU, changing the value of ‘days of stay’ from one to the actual length of stay, and then subsampled for a set of standardized days: 1, 3, 8, 15, 22, 31, 46 and 61. Some variables only applied at predetermined time intervals: Respiratory distress syndrome (0–7 days); necrotizing enterocolitis (14–45 days); intraventricular hemorrhage (0–10 days); periventricular leukomalacia (> 21 days) and bronchopulmonary dysplasia (> 28 days). With this method, we managed to bolster the training set and endow the regression module with a pseudo-evolution of all cases, enabling a time-based dynamic estimation.

#### Validation stage

In this stage, the Kappa index, Accuracy, AUC and Brier score for each model has been calculated. The Accuracy and Kappa index were calculated using the cut-off point value from the development set data, to estimate the discrete outcome (i.e. likely to live or to die). As for the Kappa index (k), strength of concordance between the predicted outcome and the true value has been analyzed with the following criteria: “Poor” k = 0–0.20; “Weak” k = 0.21–0.40; “Moderate” k = 0.41–0.60; “Good” k = 0.61–0.80; “Very Good “k = 0.81–1.00.

In all models, the prognosis was reported in categorical alternatives: "Mild: Very high probability of survival" (mortality rate: 0–20%), "Moderate: High probability of survival" (mortality rate: 21–50%), "Severe: Low probability of survival" (mortality rate: 51–80%), or "Very severe: Very low probability of survival" (mortality rate: 81–100%). For all models, we also provided cut-off points, splitting the score into four intervals using the measured False Negative Rate (FNR) as the metric to define the mortality rate thresholds. The FNR measures the number of dead cases that obtained a score smaller than the threshold, divided by the total number of dead. Since the dataset was highly biased towards non-dead individuals, if we had used the Positive Predictive Value (PPV), which assesses the estimated dead probability for each value, this might have resulted in extremely close-to-zero thresholds. Such low thresholds are non-informative when interpreting results for complex cases during the NICU stay.

The prognostic ability of Model 3 was also tested with a simulation of a “real time scenario” for different lengths of stay in the NICU: Day 1, day 8, day 15, day 31, and day 61. Throughout the validation stage we followed the same protocol applied during the development. We simulated patient profiles at different days (i.e. day 1, day 8, day 15, day 31 and day 61) and eliminated those diagnoses that were incompatible with the length of stay being tested at the time. For example, respiratory distress syndrome would only be included in those scenarios contemplating the evolution from days 0 to 7. In all cases, we previously created subsets of the validation set and applied them to patients with length of stay greater than or equal to the time point under consideration.

Finally, an online calculator to access the results of these models was developed.

## Results

14953 newborns were included, 8734 in the development stage and 6219 in the validation stage. Of the 2015 infants who died, 373 (18.5%) did so during the first 24 hours of life, 1315 (65.3%) during the first month of life and 327 (16.2%) between 30 days of life and final discharge.

Perinatal and neonatal variables, main diagnoses and severity-adjusted diagnoses during hospital admissions of both stages are shown in Table 2.

**Table 2:** Perinatal and neonatal variables, diagnoses, and diagnoses adapted according to severity criteria in the patients studied.

	Development stage	Validation stage
	(years: 2009–2012)	(years: 2013–2015)
	n = 8,734	n = 6,219
**Perinatal and Neonatal variables**		
Maternal steroids *(% no / % partial / % full)*	11.7 / 18.9 / 69.4	8.1; /18.7 / 73.3
Maternal chorioamnionitis *(%)*	18.5	21.0
Maternal hypertension (preeclampsia) *(%)*	20.1	21.7
Multiple pregnancy *(%)*	35.7	34.5
Male gender *(%)*	51.1	50.6
Gestational age (weeks and days*) (median (IQR))	29.1 (27.2–30.8)	29.1 (27.2–30.7)
Birth weight *(grams) (median; IQR)*	1,117 (885–1,320)	1,103 (870–1,320)
Low weight (<p10) *(%)*	34.4	34.2
Place of birth (% hospital)	94.5	95.8
Level of care (% level 3)	99.1	99.0
Type of delivery *(% caesarean section)*	71.1	72.9
Resuscitation in delivery room *(% nil / % positive pressure support / % intubation / % chest compressions / % adrenaline)*	35.1 / 29.0 / 29.5 / 2.6/ 3.8	31.4 / 36.4 / 26.0 / 3.0/ 3.3
Admission temperature income *(°C) (median (IQR))*	36.0 (35.4, 36.4)	36.0 (35.4–36.4)
Apgar test 1 minute *(0–10) (median (IQR))*	7 (5–8)	7 (5–8)
Apgar test 5 minutes *(0–10) (median (IQR))*	9 (8–9)	8 (7–9)
CRIB-1 *(median (IQR))*	1 (1–4)	1 (1–4)
Postnatal corticosteroids *(%)*	6.3	8.0
Death at discharge *(%)*	14.8	11.6
**Diagnoses**		
Respiratory distress syndrome *(%)*	64.0	62.1
Pneumothorax *(%)*	4.9	4.2
Patent ductus arteriosus *(%)*	35.9	33.2
Necrotizing enterocolitis *(%)*	7.7	7.2
Focal gastrointestinal perforation *(%)*	2.7	2.6
Early onset sepsis (<72 h) *(%)*	4.7	5.1
Late onset sepsis (>72 h) *(%)*	33.6	31.7
Intraventricular hemorrhage *(%)*	26.7	26.6
Periventricular leukomalacia *(%)*	6.3	7.7
Retinopathy of prematurity *(%)*	21.6	20.2
Bronchopulmonary dysplasia *(%)*	27.7	29.0
**Severity-adjusted diagnoses**		
Advanced resuscitation *(%)*	35.9	32.3
Severe hyaline membrane *(%)*	34.8	35.5
Surgery for patent ductus arteriosus *(%)*	5.6	4.8
Severe necrotizing enterocolitis *(%)*	4.9	4.9
Severe invasive infection *(%)*	16.0	14.1
Laser for retinopathy of prematurity *(%)*	4.2	3.9
Severe intraventricular hemorrhage *(%)*	9.4	8.7
Cystic periventricular leukomalacia *(%)*	2.6	2.3
Severe bronchopulmonary dysplasia *(%)*	14.5	16.6
Severe pneumothorax (%)	4.9	4.2
Severe anemia *(%)*	48.8	45.4

^a^ Gestational weeks and days are displayed as follows: 1 day = 0.14 weeks; 28 weeks and 3 days = 28.5 w.

The median GA for both periods was 29.1 weeks with a median BW of 1.117 g (IQR: 885–1320), and 1.103 g (IQR: 870–1320) in the development and validation stages, respectively. Hospital mortality was 14.8% in the development stage and 11,6% in the validation stage.

Table 3 summarizes "regression equation coefficients" and "odds ratios" of the variables with the greatest impact in mortality in each of the predictive models studied. Values of R^2^ Nagelkerke, Hosmer-Lemershow, AUC and Brier score are presented for each model.

**Table 3:** Regression coefficients, standard errors, odds ratios, and 95% confidence intervals for the multiple logistic regression equations developed for each model. The main outcome is mortality before discharge.

Variable	Regression Coefficient	Standard error	Odds Ratio	95% Confidence Interval	
**Model 1 (prenatal)**					
Intercept	16.804	0.529			
Level of care (level 2)	0.504	0.363	1.666	0.813 3.371	
Gestational age *(weeks and days)*	- 0.650	0.019	0.522	0.503 0.542	
Low weight *(<p10)*	0.988	0.085	2.685	2.272 3.173	
Male gender	0.076	0.070	1.079	0.940 1.237	
Maternal steroids (*partial)*	- 0.705	0.110	0.494	0.398 0.614	
Maternal steroids *(full)*	- 1.094	0.096	0.335	0.278 0.404	
Multiple pregnancy *(yes)*	0.299	0.074	1.348	1.167 1.558	
**Model 2 (24 hours)**					
Intercept	18.525	2.016			
Level of care (level 2)	0.726	0.415	2.066	0.877	4.491
Gestational age *(weeks and days)*	- 0.285	0.031	0.752	0.707	0.799
Male sex	0.133	0.086	1.142	0.964	1.353
Maternal steroids (*partial)*	- 0.433	0.140	0.648	0.493	0.854
Maternal steroids *(full)*	- 0.604	0.125	0.547	0.429	0.700
Birth weight *(g/100)*	- 0.274	0.026	0.760	0.722	0.800
Multiple pregnancy *(yes)*	0.315	0.091	1.370	1.146	1.638
Apgar-5 min *(0–10)*	- 0.230	0.026	0.794	0.755	0.835
Maternal hypertension *(yes)*	- 0.456	0.131	0.634	0.488	0.818
Advanced resuscitation *(yes)*	0.304	0.108	1.355	1.098	1.673
Admission temperature *(°C)*	-0.223	0.052	0.800	0.723	0.886
Severe respiratory distress syndrome *(yes)*	0.329	0.093	1.390	1.159	1.668
**Model 3 (0–30 days of life)**					
Intercept	6.627	0.379			
Multiple pregnancy *(yes)*	0.297	0.049	1.345	1.222	1.480
Gestational age *(weeks and days)*	-0.163	0.016	0.850	0.824	0.876
Maternal steroids *(yes)*	-0.306	0.067	0.736	0.646	0.840
Maternal hypertension *(yes)*	-0.367	0.069	0.693	0.605	0.792
Apgar-5 min *(0–10)*	-0.198	0.013	0.820	0.800	0.841
Birth weight *(g/100)*	-0.322	0.013	0.725	0.706	0.744
Severe respiratory distress syndrome *(yes)*	0.132	0.056	1.141	1.022	1.273
Severe pneumothorax *(yes)*	0.859	0.080	2.362	2.018	2.761
Necrotizing enterocolitis *(yes)*	1.584	0.086	4.874	4.112	5.771
Severe invasive infection (*yes)*	1.083	0.048	2.952	2.690	3.240
Intraventricular hemorrhage *(yes)*	0.470	0.067	1.600	1.403	1.822
Severe intraventricular hemorrhage *(yes)*	1.551	0.079	4.718	4.045	5.509
Days of life	-0.049	0.0034	0.952	0.945	0.958
**Model 3 (> 30 days of life)**					
Intercept	2.097	0.875	8.142		
Multiple pregnancy *(yes)*	0.177	0.098	1.194	0.984	1.445
Gestational age *(weeks)*	-0.190	0.033	0.827	0.775	0.881
Maternal steroids *(yes)*	-0.291	0.128	0.748	0.585	0.965
Apgar-5 min *(0–10)*	-0.152	0.024	0.859	0.819	0.901
Birth weight *(g/100)*	-0.174	0.028	0.840	0.796	0.887
Severe pneumothorax *(yes)*	0.529	0.157	1.698	1.236	2.292
Necrotizing enterocolitis *(yes)*	1.393	0.199	4.028	2.692	5.888
Severe Necrotizing enterocolitis	0.130	0.218	1.139	0.750	1.762
Severe invasive infection *(yes)*	1.478	0.106	4.388	3.574	5.415
Severe Intraventricular hemorrhage *(yes)*	0.556	0.118	1.743	1.378	2.193
Cystic Periventricular leukomalacia *(yes)*	0.655	0.147	1.925	1.437	2.557
Severe anemia *(yes)*	0.535	0.206	1.708	1.159	2.602
Bronchopulmonary dysplasia *(yes)*	1.187	0.162	3.279	2.405	4.552
Days of life	-0.004	0.0022	0.996	0.992	1.001

**- Model 1:** R^2^ Nagelkerke = 0.329; Hosmer Lemershow, p = 0.239; AUC = 0,834 (95%CI: 0.822–0.846); Brier score = 0.09.

**- Model 2:** R^2^ Nagelkerke = 0.405; Hosmer Lemershow, p = 0.125; AUC = 0.872 (95%CI: 0.860–0.884); Brier score = 0.081.

**- Model 3 (≤30 days):** R^2^ Nagelkerke = 0.448; Hosmer Lemershow p = < 2e-16; AUC = 0.989 (95%CI: 0.983–0.996); Brier score = 0.049.

**- Model 3 (>30 days):** R^2^ Nagelkerke = 0.330; Hosmer Lemershow p = 0.09; AUC = 0,942 (95%CI: 0.929–0.956); Brier score = 0.016.

In the development stage, AUC was 0.834 (95% CI: 0.822–0.846) (p<0.001) in model 1 and 0.872 (95% CI: 0.860–0.884) (p<0.001) in model 2. In model 3, AUC was 0.989 (95% CI: 0.983–0.996) (p<0.001) and 0.942 (95% CI: 0.929–0.956) (p<0.001) during the 0–30 and >30 days of life, respectively. The AUC of CRIB-1 during the first 12 hours of life to predict mortality was 0.851 (95% CI: 0.832–0.859) (p<0.001).

Predictive values obtained for cut-off points for the different models, concordance (Kappa index), and accuracy in the validation stage are shown in Table 4.

**Table 4:** Validation of predictive formulas and summary of cut-off points of the best models according to FNR (false negative rate).

Predictive Model	Number of Patients	Kappa index	Accuracy (%)	AUC	Brier Score	Cut point	FNR threshold–severity prognosis			
							**0–20%**	**21–50%**	**51–80%**	**81–100%**
Model 1 (prenatal)	6,124 (*704 died)*	0.447	87.8	0.860	0.077	0.33	0–0.158	0.159–0.368	0.369–0.567	0.568–1
Model 2 (24 hours)	5,410 *(610 died)*	0.475	89.20	0.867	0.071	0.38	0–0.169	0.170–0.420	0.421–0.649	0.650–1
Model 3 (During admission)	5,292 *(544 died)*	0.718	93.38	0.977	0.090	≤ 30 days: 0.0155	0–0.146	0.147–0.418	0.419–0.746	0.747–1
						> 30 days: 0.092	0–0.027	0.0278–0.114	0.115–0.290	0.291–1

**Kappa Index** (Force of concordance): Poor (0–0.20), Weak (0.21–0.40) Moderate (0.41–0.60), Good (0.61–0.80), Very Good; 0.81–1. **AUC** (Area under the curve). **Brier score** (Accuracy of the prognostic model prediction): From 0 for a perfect model to 0.25 for a non-informative model. **Cut-off point** was determined using only the training set data and was aimed at maximizing the Kappa index value. The Accuracy and Kappa index was calculated using the cut-off point value.

In the validation stage, models 1 and 2 showed a moderate concordance(Kappa index of 0.447 and 0.475 respectively), while for model 3, it was good (Kappa index of 0.718). Table 4 also includes additional columns that define cut-off points, splitting the score into four intervals for prognostic interpretation. The thresholds have been defined using the false negative ratio (FNR) as the metric and are aimed to group the prognoses in four groups: Very high, high, low and very low probability of survival.

We also analyzed the prognostic ability of Model 3 in a 'real time scenario' in which we evaluated the validation set, imposing a specific value for the days of life. The following values of AUC were obtained in different lengths of stay: AUC of 0.912; 0.916; 0.919; 0.923 and 0.843 on days 1, 8, 15, 31 and 61, respectively, with an overall unweighted average of AUC of 0.903.

The three predictive models are available online for educational purposes at: https://mbosio85.github.io/SEN_calc/ [22].

## Discussion

Multivariate prediction models are used to predict diagnoses or certain outcomes for a specific group of patients. When applied to a single patient, these models provide individualized prognostic information. When applied to groups of patients, predictive models allow group stratification for clinical trials, as well as case adjustment for quality improvement projects [3].

An excellent systematic review of mortality predictive models for very preterm infants was published in 2011 [3]. It included 41 studies, with 50 models to be applied either prenatally or at different times during NICU admission. After this publication, at least 8 new predictive models have been described [23–30] in a recent systematic review [31]. The most commonly used mortality predictive models today include CRIB I and II [4,32] (year 1993 / United Kingdom—first 12 hours of life; 2003 / United Kingdom—first hour of life), DRAPER [6] (2000 / United Kingdom—childbirth start and admission to NICU), SNAP II and SNAPPE II [7] (2001 / Canada—first 12 hours of life), NEOCOSUR [33] (2005 / South America—before admission to NICU), NICHD models [5,24] (2008 / USA—at the beginning of mechanical ventilation; 2012 / USA- sequential: at birth, 7th day of life, 28 days of life and 36 weeks post-menstrual), SAW [34] (2008 / Egypt—48 hours of life) and PREM Score [35] (2010 / United Kingdom—prenatal and birth). Limitations of the existing predictive models are: Development before the widespread use of surfactant [4,6], data collected up to 15 years before publication date [24,26,28,29], or no existing external validation [3]. In Spain, predictive models for preterm infants based on local population data do not exist. Moreover, the two most frequently used models in clinical practice (CRIB and the NICHD-2008 model) have not been validated in very preterm Spanish infants.

The Spanish SEN1500 network has generated population studies on extreme premature patient mortality [10,15,16], the impact on morbidity and mortality at the extreme thresholds of resuscitation (22–25 weeks of gestation) [13,14], and associated morbidity during hospital admission [16,17]. Within the SEN1500 network population, the CRIB score showed a better prediction of death when compared to GA, whereas the prediction of severe intraventricular hemorrhage did not differ according to the predictor used [16].

The possibility of using different groups of patients from the SEN1500 database has been essential for the development of the three predictive models described in this paper. What’s more, having a short interlude between the two stages, similar to what was done in the Canadian model [25], enhances the relevance of our study in comparison to the rest of studies mentioned in this discussion, that were developed with older data.

At the moment of regression, the GLM method was preferred given its ability to unify the treatment of numerical and categorical variables into proxy numerical encoding. Although the calculator was designed to offer a more accurate prediction, prognosis is also expressed in four categories; very high, high, low and very low survival probability, with a prediction of death of 0–20%, 21–50%, 51–80%, and 81–100%, respectively. Such categories were designed to help interpret the results and guide the clinician in the communication with the family, avoiding the reliance on a sole numeric value for the prediction. Moreover, it also considers the non-linearity of the actual diagnostic process, and takes into account a margin for doubt when making hard decisions that may not be backed by enough numerical precision. The moderate and severe categories were introduced to inform the doctor when the model lacks certainty to set a clear prediction. Caution should be exercised in this process as it may have severe repercussions for both patients and families.

In model 3, length–of stay was included. We are aware that time has a non-linear influence on mortality, and that we do not have the ideal scenario with time-based information (e.g. on which day a patient acquired a disease, or recovered from it). However, we believe that even a linear approximation is useful to generate dynamic predictions, and might favor continuous data gathering and processing to help improve outcome precision.

In all predictive models of this study, hospital mortality was used exclusively as a dependent variable without including other variables, such as disability or neurological injury, as described in other publications [5,24,28,36]. Our developed models showed good results in the AUC and accuracy (Brier score) in predicting mortality, especially model 3 during the first month of life. In addition, model 3 showed the highest concordance when validated (Kappa = 0.718; Accuracy = 93.38%). Model 2 obtained an AUC higher than CRIB-1.

Some of the variables included in this study (respiratory distress syndrome, intraventricular hemorrhage, necrotizing enterocolitis, etc), have been expressed in two ways; absolute terms (yes/no) and severity (mild/severe). Even though they are not mutually exclusive, they add depth to patient status, improving the final outcome. For example, having a mild form of intraventricular hemorrhage has a different effect on outcome, when compared to a more severe degree. If we include IVH as a 3-category variable (no IVH, mild IVH, or severe IVH), order would be disregarded. Conversely, if it is incorporated as a simple numerical variable, the model grants the same relevance to both the gap from having no IVH up to a mild form of IVH, and the gap from having mild IVH up to severe IVH. Therefore, by combining the 2 systems, we bestow the model with a cumulative effect, thus preserving the freedom to estimate differently the increase of mortality according to the degree of severity.

In model 1 (prenatal), younger GA, non-administration of maternal steroids, BW below 10^th^ centile, multiple pregnancy, and non-tertiary level of care were the variables with the greatest impact on hospital mortality. In contrast, male gender had a minor impact compared to other studies [5,24]. This is important for regionalized maternal care and maternal transport, as strategic interventions can improve neonatal outcomes.

In model 2 (first 24 hours of life), lower BW, multiple pregnancy, lower Apgar at 5 minutes, maternal hypertension, advanced resuscitation, lower admission temperature, severe respiratory distress syndrome, and the significant variables of model 1 were the variables that had the greatest impact on hospital mortality. Interestingly, our analysis demonstrated a protective effect of maternal hypertensive disorders on the risk of infant mortality. This association has also been identified in a 2016 cohort study published by L. Gemmell et al, in which premature infants born at 24–28 weeks' gestation to mothers with hypertensive disorders had a lower risk of mortality [37]. It has been hypothesized that maternal hypertension is an adaptive response that accelerates organ maturation in a fetus under stress, and could explain the lower mortality rate observed in this patient population. In other predicted models, the time period in which predictions were made included; the moment of birth [19,27], admission to NICU [6] or the first 12 hours of life [4,7]. In our project, we chose the first 24 hours of life, to better encompass the effect of severe respiratory distress syndrome (administration of surfactant and the use of mechanical ventilation), which increases mortality in the same way as the Canadian model [25] that also includes the first 24 hours of life.

In model 3, we chose a double stratified model by days of hospital stay (0–30 days and >30 days) because it had shown greater accuracy when compared to a single model in previous analyses. This fact may derive from a different impact of neonatal variables on mortality in the first weeks of life compared to later on. Apart from days of life, the other included variables in model 3 (0–30 days) are those from model 2 (with the exception of level of care, advanced resuscitation and admission temperature), with the addition of other potentially significant morbidity variables linked to that time period. The aforementioned variables would be; severe pneumothorax, necrotizing enterocolitis, severe invasive infection and intraventricular hemorrhage, especially grades 3–4. After 30 days of life, severe respiratory distress syndrome or HIV become less important as predictors of death, in comparison to the more chronic conditions, such as cystic periventricular leukomalacia, severe anemia and BPD (considered as needing additional oxygen or respiratory support at 36 weeks of corrected age). This double stratified model by days of hospital stay reflects such effects.

In another predictive model published by Ambalavanan N et al [24], different times have been used as predictors of mortality apart from birth (7 and 28 days of life, and 36 weeks of postmenstrual age) [24]. Although FiO_2_ was the main predictor for death at all specified postnatal time points in that model, other variables had a leading influence on death in different time periods (for instance, BW and intraventricular haemorrhage at 7 days of age). At 28 days of age, there would be the number of clinical late-onset infections and days of parenteral nutrition. Finally, at 36 weeks postmenstrual age, other variables that predominantly influence death would be the total number of late-onset sepsis episodes, a higher number of days on ventilation, and having enlarged ventricles.

In our study, another cohort of patients, born in 2013–15, was used to validate the three predictive models developed using the preterm population of years 2009–2012. Furthermore, as validation measures, AUC, accuracy and Kappa index were calculated. This was the chosen methodology since we believe that it presents a better alternative to bootstrapping the same population in a later period, improving implementation [38].

The strengths of the developed models would be the use of a large and recent population and having good levels of AUC in the mortality prediction, especially model 3 (during admission), which showed an AUC of 0.977 and a good concordance (Kappa index of 0.718). Additionally, the existence of an online tool allows better implementation in daily clinical practice, as described by other authors [5,24,29].

On the down side, there are some major limitations in this model. First, the absence of family perspectives in the model design, already proposed by some authors [39]. Second, an external validation of this model in other groups of patients has not yet been carried out. Third, all models designed with existing data run the risk of having a self-fulfilling prophecy effect. Finally, there are no publications concerning the use of these types of models by families and clinicians, that we know of.

In conclusion, three predictive mortality models were developed and validated for the Spanish population of very preterm infants. We showed that the use of dynamic models in individual mortality can improve outcome predictions. Validation studies testing our online calculator in different settings and prospective populations are needed in order to confirm its accuracy and generalizability.

## Supporting information

S1 Data (SAV)

## Abbreviations

AUC: area under the curve
BPD: bronchopulmonary dysplasia
BW: birth weight
CPAP: continuous positive airway pressure
FNR: false negative rate
GA: gestational age
GLM: generalized linear model
IPPV: intermittent positive-pressure ventilation
NEC: necrotizing enterocolitis
NICU: Neonatal Intensive Care Unit
NPV: negative predictive value
PDA: patent ductus arteriosus
PPV: positive predictive value

## References

[1] Pallas Alonso CR, Arriaga Redondo M. Nuevos aspectos en torno a la prematuridad. Evid Pediatr 2008; 4:26.

[2] Debray TP; Damen JA, Snell KI, Ensor J, Hooft L, Reitsma JB, et al A guide to systematic review and meta-analysis of prediction model performance. BMJ 2017;356:1–11. 10.1136/bmj.i6460 28057641

[3] Medlock S, Ravelli ACJ, Tamminga P, Mol BWM, Abu-Hanna A. Prediction of Mortality in Very Premature Infants: A Systematic Review of Prediction Models. PLoS ONE (2011) 6(9): e23441 10.1371/journal.pone.0023441 21931598 PMC3169543

[4] Cockburn F, Cooke RWI, Gamsu HR et al The CRIB (clinical risk index for babies) score: a tool for assessing initial neonatal risk and comparing performance of neonatal intensive care units. Lancet 1993; 342: 193–8. 8100927

[5] Tyson JE, Parikh NA, Langer J, Green C, Higgins RD; National Institute of Child Health and Human Development Neonatal Research Network. Intensive care for extreme pre- maturity—moving beyond gestational age. N Engl J Med 2008;358:1672–81. 10.1056/NEJMoa073059 18420500 PMC2597069

[6] Draper ES, Manktelow B, Field DJ, James D. Prediction of survival for preterm births. BMJ 2000;321:237. PMC1118228 10979676

[7] Richardson DK, Corcoran JD, Escobar GJ, Lee SK. SNAP-II and SNAPPE-II: Simplified newborn illness severity and mortality risk scores. J Pediatr 2001;138: 92–100. 10.1067/mpd.2001.109608 11148519

[8] INE. http://www.ine.es/jaxi/Datos.htm?path=/t20/e301/nacim/a2008/l0/&file=01011.px (Access on December 22, 2018)

[9] INE. http://www.ine.es/jaxi/Datos.htm?path=/t20/e301/nacim/a2017/l0/&file=01011.px (Access on December 22, 2018)

[10] Moro M, Figueras-Aloy J, Fernández C, Doménech E, Jiménez R, Pérez-Rodríguez J et al Vicente and Grupo SEN1500. Mortality for new-borns of birthweight less than 1500 g in Spanish neonatal units (2002–2005) Am J Perinatol 2007; 24: 593–601. 10.1055/s-2007-992175 17972231

[11] Moro M, Fernández C, Figueras-Aloy J, Pérez-Rodríguez J, Coll E, Doménech E et al Grupo SEN1500.SEN1500: diseño y desarrollo del registro de niños de menos de 1.500 g al nacer en España. An Pediatr (Barc) 2008;68:181–8. 18341886 10.1157/13116235

[12] García P, San Feliciano L, Benito F, García R, Guzmán J, Salas S et al Seguimiento a los 2 años de edad corregida de una cohorte de recién nacidos con peso inferior o igual a 1.500 g de los hospitales pertenecientes a la red neonatal SEN1500. An Pediatr (Barc) 2013;79:279–87. 23684170 10.1016/j.anpedi.2013.03.017

[13] García-Muñóz RF, García-Alix Pérez A, García Hernández JA, Figueras Aloy J y Grupo español SEN1500. Morbimortalidad en recién nacidos al límite de la viabilidad en España: estudio de base poblacional. An Pediatr (Barc) 2014; 80:348–56. 24560497 10.1016/j.anpedi.2013.12.012

[14] García-Muñóz RF, Díez Recino A.L, García-Alix Pérez A, Figueras Aloy J, Vento Torres M and the SEN1500 Network. Changes in Perinatal Care and Outcomes in Newborns at the Limit of Viability in Spain. The EPI-SEN Study. Neonatology 2015;107:120–9. 10.1159/000368881 25502078

[15] Figueras J, Moro M, Perez-Rodriguez J, Fernandez C, Roques V, Quero J, et al Antenatal glucocorticoid treatment decreases mortality and chronic lung disease in survivors among 23–28 w gestational age preterm infants. Am J Perinatol 2005;22:441–8. 10.1055/s-2005-916332 16283604

[16] Guzman J, Parraga Mj, Del Prado N, Ruiz Md, Garcia Del Rio M, Benito F, et al Análisis de la utilidad del CRIB por estratos de peso como predictor de muerte hospitalaria y de hemorragia intraventricular grave en España. An Pediatr (Barc) 2009;71:117–27. 19595649 10.1016/j.anpedi.2009.04.007

[17] Moro M, Perez-Rodriguez J, Figueras J, Fernandez C, Domenech E, Jimenez R, et al Predischarge morbidities in extremely and very-low-birth-weight infants in Spanish Neonatal Units. Am J Perinatol 2009;26:335–43. 10.1055/s-0028-1110083 19090453

[18] McHugh M. Interrater reliability: the kappa statistic. Biochemia Medica 2012;22:276–282. 23092060 PMC3900052

[19] Hosmer D, Lemeshow S, Sturdivant R. Applied Logistic Regression. 3rd ed. Wiley 2013 Pag 157.

[20] Brier G. Verification of Forecasts expressed in terms of probability. Monthly Weather Review 1950;78(1):1–3.

[21] Rufibach K. Use of Brier score to assess binary predictions. Journal of Clinical Epidemiology 2010;63(8):938–939. 10.1016/j.jclinepi.2009.11.009 20189763

[22] SEN1500 Online Calculator: https://mbosio85.github.io/SEN_calc/.

[23] Shah PS, Ye XY, Synnes A, Rouvinez-Bouali N, Yee W, Lee SK et al Prediction of survival without morbidity for infants born at under 33 weeks gestational age: A user-friendly graphical tool. Arch Dis Child Fetal Neonatal Ed 2012;97:F110–5 10.1136/archdischild-2011-300143 21900280

[24] Ambalavanan N, Carlo WA, Tyson JE, Langer JC, Walsh MC, Parikh NA et al Outcome trajectories in extremely preterm infants. Pediatrics 2012;130:e115–25. 10.1542/peds.2011-3693 22689874 PMC3382921

[25] Ge WJ, MIrea L, Yang J, Bassil KL, Lee SK, Shah PS et al Prediction of neonatal outcomes in extremely preterm neonates. Pediatrics 2013;132:e876–85. 10.1542/peds.2013-0702 24062365

[26] Ravelli AC, Schaaf JM, Mol BW, Tamminga P, Eskes M, van der Post JA et al Antenatal prediction of neonatal mortality in very premature infants. Eur J Obstet Gynecol Reprod Biol 2014;176:126–31. 10.1016/j.ejogrb.2014.02.030 24666798

[27] Márquez-González H, Jiménez-Báez MV, Muñoz-Ramírez CM, Yáñez-Gutierrez L, Huelgas-Plaza AC, Almeida-Gutierrez E et al Development and validation of the neonatal mortality score-9 Mexico to predict mortality in critically ill neonates. Arch Argent Pediatr 2015;113:213–20. 10.5546/aap.2015.213 25996319

[28] Schmidt B, Roberts RS, Davis PG, Doyle LW, Asztalos EV, Opie G et al Prediction of late death or disability at age 5 years using a count of 3 neonatal morbidities in very low birth weight infants. J Pediatr 2015;167:982–6. 10.1016/j.jpeds.2015.07.067 26318030

[29] King CP, da Silva O, Filler G, Lopes LM. Online calculator to improve counselling of short term neonatal neonatal morbidity and mortality outcomes at extremely low gestational age (23–28 weeks) Am J Perinatol 2016;33:901–7. 10.1055/s-0036-1581131 27057769

[30] Podda M, Bacciu D, Micheli A, Bellù R, Placidi G, Gagliardi L. A machine learning approach to estimating preterm infantssurvival: Development of the Preterm Infants Survival Assessment (PISA) predictor. Scientific Reports 2018;8:12743, 10.1038/s41598-018-31122-0 30213963 PMC6137213

[31] Del Río R, Thió M, Bosio M, Figueras-Aloy J, Iriondo M. Mortality Prediction of mortality in premature neonates. An updated systematic review. An Pediatr (Barc) (article in press). 10.1016/j.anpedi.2019.11.003 31926888

[32] Parry G, Tucker J, Tarnow-Mordi W, the UK Neonatal Staffing Study Collaborative Group. CRIB-II: an update of the clinical risk index for babies score. Lancet 2003; 361: 1789–91. 10.1016/S0140-6736(03)13397-1 12781540

[33] Marshall G, Tapia JL, D´Apremont I, Grandi C, Barros C, Alegria A et al A new score for predicting neonatal very low birth weight mortality risk in the Neocosur South American Network. J Perinatol 2005;25:577–82. 10.1038/sj.jp.7211362 16049510

[34] Rosenberg RE, Ahmed S, Saha SK, Ahmed AS, Chowdhury MA, Law PA, et al Simplified age-weight mortality risk classification for very low birth weight infants in low-resource settings. J Pediatr.2008 10;153(4):519–24. 10.1016/j.jpeds.2008.04.051 Epub 2008 Jun 9. 18539298

[35] Cole TJ, Hey E, Richmond S: The PREM score: a graphical tool for predicting survival in very preterm births. Arch Dis Child Fetal Neonatal Ed 2010;95:F14–9. 10.1136/adc.2009.164533 19700396

[36] Boland RA, Davis PG, Dawson JA, Doyle LW, for the Victorian Infant Collaborative Study Group. Predicting death or major neurodevelopmental disability in extremely preterm infants born in Australia. Arch Dis Child Fetal Neonatal Ed 2012;0:F1–4. 10.1136/archdischild-2012-301628 23134711

[37] Gemmell L, Martin L, Murphy KE, Modi N, Håkansson S, Reichman B, et al Hypertensive disorders of pregnancy and outcomes of preterm infants of 24 to 28 weeks’ gestation. J Perinatology 2016; 36, 1067–1072. 10.1038/jp.2016.133 27583388

[38] Efron B., Tibshirani R. Bootstrap Methods for Standard Errors, Confidence Intervals, and Other Measures of Statiscal Accuracy. Statistical Science 1986; 1: 54–77.

[39] Bell EF, Rysavy MA. What parents want to know after preterm birth. J Pediatr 2018;200:10–1. 10.1016/j.jpeds.2018.04.025 29752178

