# Correction: Prediction of mortality in very low birth weight neonates in Spain

**DOI:** 10.1371/journal.pone.0285353

**Published:** 2023-05-01

**Authors:** Martín Iriondo, Marta Thio, Ruth del Río, Benjamin J. Baucells, Mattia Bosio, Josep Figueras-Aloy

The following information is missing from the Funding statement: This study has been funded by Instituto de Salud Carlos III through the project "PI16/00748" (Co-funded by European Regional Development Fund/European Social Fund "A way to make Europe"/"Investing in your future") awarded to MI.
